# Acitretin Treatment for Lipoid Proteinosis

**DOI:** 10.1155/2012/324506

**Published:** 2012-08-09

**Authors:** Özgür Gündüz, Neriman Şahiner, Pınar Atasoy, Çağrı Şenyücel

**Affiliations:** ^1^Department of Dermatology and Venerology, Suleyman Demirel Teaching Hospital, University of Kırıkkale, Fabrikalar Mahallesi, Saglık Sokak, Kırıkkale 71100, Turkey; ^2^Department of Pathology, School of Medicine, Kırıkkale University, Kirikkale 71100, Turkey; ^3^Department of Radiodiagnostics, School of Medicine, Kırıkkale University, Kirikkale 71100, Turkey

## Abstract

Lipoid proteinosis (LP) is a rare, autosomal-recessive disease characterized by the hoarseness and widespread cutaneous scarring, more prominent on sun-exposed areas. Yellow-white plaques can be seen on oral mucosa and on the skin among depressed scars. Histological evaluation of the affected sites shows accumulation of hyaline-like material in dermis and disruption of basement membrane. Although LP is compatible with normal life expectancy, involvement of upper respiratory tract may endanger patient's life, especially in the case of a respiratory tract infection. Involvement of central nervous system has also been reported, but its clinical importance is obscure. Due to the rarity of LP, a definite therapeutical approach is not established. In this paper we describe a 21-year-old LP patient who was treated with acitretin for six months. Although the outcome with cutaneous lesions was not satisfactory, her hoarseness was significantly improved.

## 1. Introduction

LP, first described in 1929 [1], is a rare, progressive autosomal-recessive disease. As of today, approximately 300 cases are reported in the literature. Although in the 1970s South Africa was known to have the largest group of LP patients [2], most of the recently reported case series are from the Mediterranean or Middle Eastern countries like Turkey [3], Tunisia [4], and Saudi Arabia [5]. Skin and upper respiratory tract mucosa are the most prominently affected sites. Common clinical manifestations of LP are weak cry and hoarseness in infancy and widespread scarring and infiltrated plaques on mucosal surfaces and skin, especially at the sites of minor trauma and on sun-exposed areas, later in life. Histopathological evaluation of lesional biopsies shows accumulation of hyaline-like material in dermis and disruption of basement membrane, characteristic histopathological features for LP [6]. Although various therapeutic approaches have been suggested for the treatment, results are mostly conflicting and a definite therapy regimen has yet to be established.

## 2. Case History

**Figure 1:**
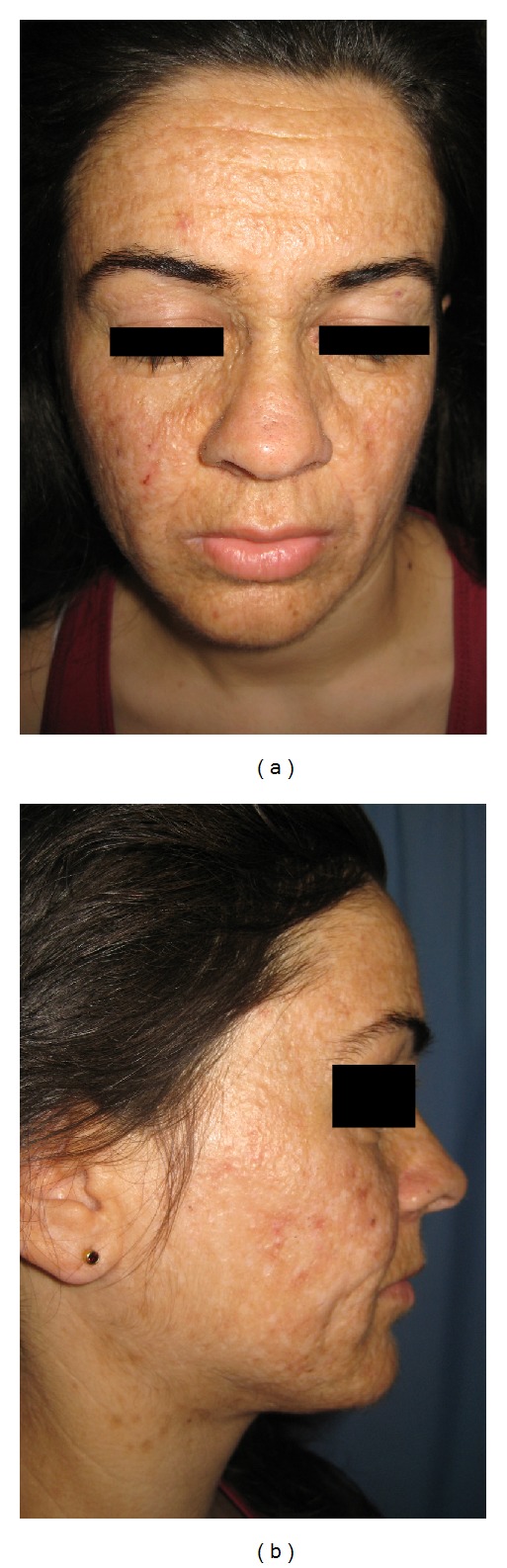
(a) Widespread, indurated flat papules and plaques. (b) Depressed acneiform scars more prominent on malar area.

**Figure 2:**
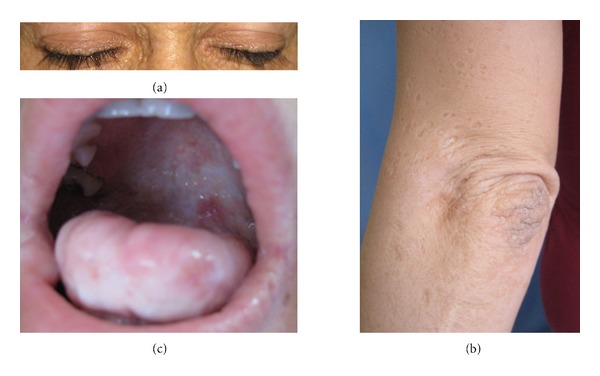
(a) Classic beaded eyelid papules-indurated papules at the base of eyelashes. (b) Pock-like scars on the left arm and hyperkeratotic, verrucous plaque on the left elbow. (c) Yellow infiltrated plaques on hard palate and under the tongue.

**Figure 3:**
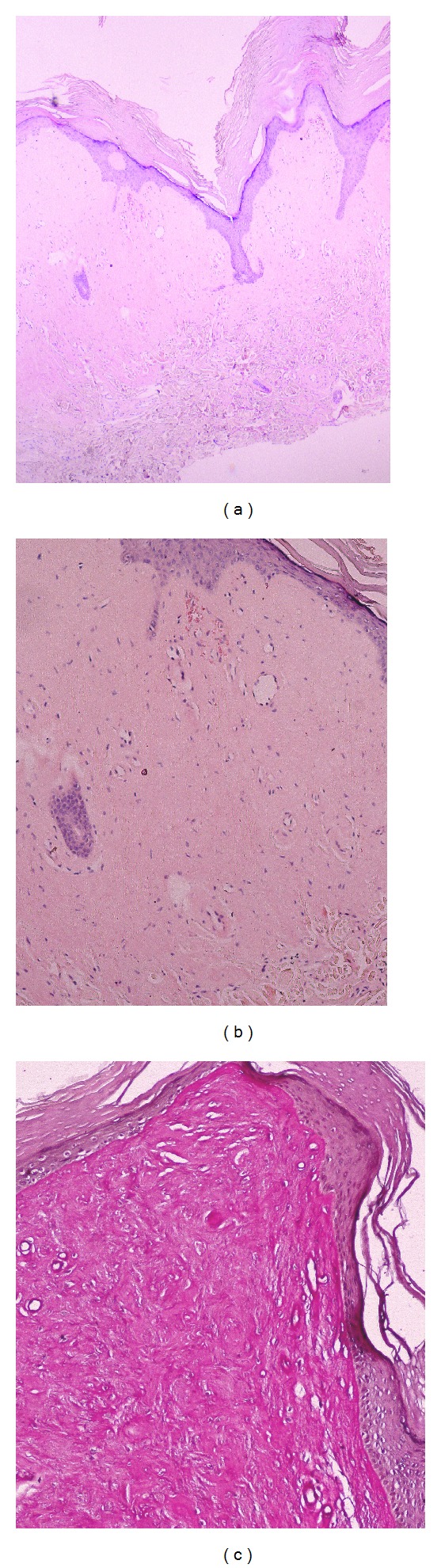
Histological features of LP (a) Epidermal hyperkeratosis and accumulation of hyaline material in dermis (H and E x 40). (b) A skin biopsy was performed from the hyperkeratotic plaque on the right elbow, revealing epidermal hyperkeratosis, eosinophilic, amorphous, infiltrate throughout the dermis and thickening of basement membrane (Figures 3(a) and 3(b)). The infiltrate showed strong staining with periodic-acid Schiff (PAS (+)) (Figure 3(c)).

**Figure 4:**
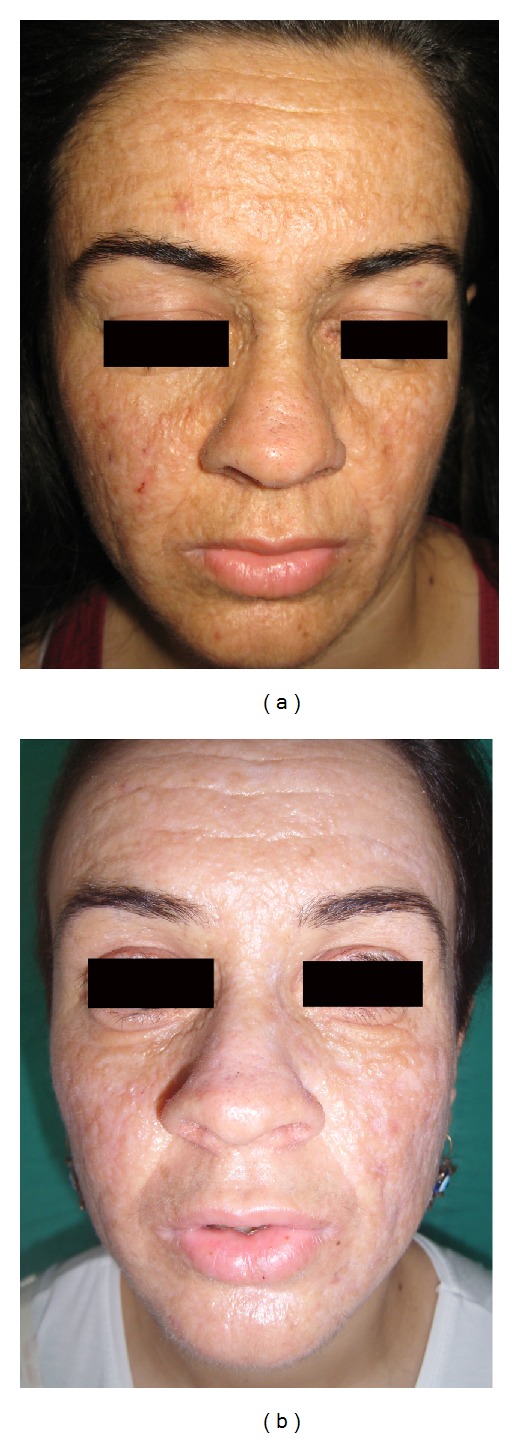
Comparison between the start (a) and end of acitretin therapy (b). After 6 months, improvement of cutaneous lesions was not satisfactory, but there was significant improvement of hoarseness.

A 21-year-old female presented with diffuse, facial papules, plaques, and atrophic scars. Initial scarring had begun on her face six years ago and then progressed to her shoulders and arms. In addition, she had a history of weak cry during infancy and hoarseness since birth. During dermatological examination, yellowish tinge and waxy texture of her face were noted. Extensive atrophic scars, glossy, infiltrated, yellow papules, and plaques were present on her forehead and cheeks (Figures 1(a) and 1(b)) and beaded papules on the margins of the upper eyebrows (Figure 2(a)). Similar scars were found on her shoulders, around her elbows, and on the back of her hands (Figure 2(b)). Yellow infiltrated plaques were covering the mucosa on the hard palate, ventral surface of the tongue, and the floor of her mouth, limiting tongue movement (Figure 2(c)). Laryngoscopy revealed thickening of the vocal cords. Evaluation of central nervous system with magnetic resonance imaging and tomography showed parenchymal calcifications in hippocampal and parahippocampal areas. A skin biopsy was performed from the hyperkeratotic plaque on the right elbow, revealing epidermal hyperkeratosis, eosinophilic, amorphous, infiltrate throughout the dermis, and thickening of basement membrane (Figures 3(a) and 3(b)). The infiltrate showed strong staining with periodic-acid Schiff (PAS (+)) (Figure 3(c)). LP diagnosis was established and the patient was started on acitretin with a dose of 25 mg/day (app. 0.5 mg/kg/day). After six months, the cutaneous plaques have become less indurated. The most striking outcome of the acitretin therapy was the significant improvement of the hoarseness. Our patient objected to undergo a second laryngoscopy and was lost to our followup at the end of six months. One year after the end of the therapy, she revisited our clinic. All her cutaneous lesions were still present (Figure 4) and improvement of the hoarseness was deteriorated.

## 3. Discussion

Lipoid proteinosis (LP), also known as hyalinosis cutis et mucosae or Urbach-Wiethe disease, is a rare, autosomal-recessive disorder. It was first described in 1929 [1] by the Viennese dermatologist Urbach and otorhinolaryngologist Wiethe and named after the histological findings, which was thought to reflect abnormal protein and lipid deposition in various tissues. 

Weak cry and hoarseness are usually initial symptoms. Cutaneous lesions may not occur until after 10 years of age [2], making the diagnosis in children difficult. LP may also manifest with vesiculobullous lesions, oral ulcers, and anemia [7, 8]. 

Skin lesions are more prominent on sun-exposed areas, especially on face. Diffusely thickened facial skin usually has a waxy texture and is covered with widespread yellow infiltrated, flat papules, and plaques among depressed scars. Verrucous plaques and atrophic scars are also evident on elbows and hands. Hu et al. reported an extraordinary case of possible LP with solely hand involvement [9]. 

Oral mucosa is invariably the initial affected site. Besides hoarseness and restricted tongue protrusion due to vocal chord and frenulum infiltration, respiratory distress attacks can occur, when the partially obstructed airway is infected [10].

LP also affects extracutaneous tissues, such as central nervous system (CNS). Central nervous system involvement can usually be observed in the form of calcified spots in the temporal lobes or hippocampus amygdala. Epileptic seizures are reported in about 25% of LP patients but correlation between seizures and intracranial calcifications is not established [11].

Histolopathological evaluation of affected sites shows periodic-acid Schiff (PAS) positive, diastase-resistant thickening of the basement membrane at the dermal-epidermal junction surrounding vessels and adnexa, and accumulation hyaline material in dermis [12]. 

Until within the past decade, the etiology of LP has been obscure. In 2002, Hamada et al. demonstrated loss-of-function mutations in the extracellular matrix protein 1 gene (ECM1) of LP patients [13],located on chromosome 1q21 next to the epidermal differentiation complex [14]. ECM1 protein is expressed in various tissues including epidermis and dermis [15]. Interaction between ECM1 protein and perlecan, a major proteoglycan of basement membrane, has been recognized in recent years [16, 17] and circulating autoantibodies against ECM1 have been found in the sera of the patients with lichen sclerosus (LS) [18], which is another dermatological disease with some overlapping histopathological features with LP (hyperkeratosis, hyalinization in upper dermis). All these histological studies and abnormalities implicated in LP and LS suggest possible roles for ECM1 in epidermal differentiation, formation of cutaneous basement membrane zone and dermis, and indicate the loss of ECM1 function as the possible cause of clinical and histological features of LP.

Due to the rarity of LP, there are no large case series to evaluate the therapeutic options. Anecdotal good results have been reported with oral dimethyl sulphoxide (DMSO) [19], D-penicillamine [20], etretinate [21], and acitretin [22–24], and carbon dioxide laser surgery has been proposed in the treatment of affected vocal cords [25] and eyelid papules [26]. However there are also reports of limited success [22, 23] or unsatisfactory results with some of these medications [24, 27]. Experience with acitretin in LP patients is limited [22–24] and conflicting results have been reported. Retinoids are proposed to modulate the metabolism of basement membrane [28]. Hein et al. reported the inhibitory effects of vitamin A derivatives on collagen production [29]. Via this inhibitory effect, acitretin may decrease the deposition of hyaline material in dermis and restore the basement membrane. This inhibitory effect was the underlying rationale for choice of acitretin. In our case, we have not observed any obvious cutaneous improvement in our patient, but there was significant improvement in her hoarseness. A similar outcome was first reported by Toosi and Ehsani [22] in a 3-year-old girl with LP. Since there is limited experience with acitretin, large case series are needed to evaluate the effects of acitretin on LP patients, but recent case reports, including ours, indicate a possible role for acitretin in the treatment of mucosal LP. 

Despite the variety of the reported treatment options for LP, there have been no lasting improvements or total cure. Since the association between ECM1 mutations and LP has been revealed, more effective therapeutic interventions including recombinant gene therapies may be developed in the near future.
